# Accelerating Kinetics with Time-Reversal Path Sampling

**DOI:** 10.3390/molecules28248147

**Published:** 2023-12-18

**Authors:** Zhirong Liu

**Affiliations:** Beijing National Laboratory for Molecular Sciences (BNLMS), College of Chemistry and Molecular Engineering, Peking University, Beijing 100871, China; liuzhirong@pku.edu.cn

**Keywords:** accelerated kinetics, nonequilibrium statistics, time reversibility, enhanced sampling, protein folding

## Abstract

In comparison to numerous enhanced sampling methods for equilibrium thermodynamics, accelerating simulations for kinetics and nonequilibrium statistics are relatively rare and less effective. Here, we derive a time-reversal path sampling (tRPS) method based on time reversibility to accelerate simulations for determining the transition rates between free-energy basins. It converts the difficult uphill path sampling into an easy downhill problem. This method is easy to implement, i.e., forward and backward shooting simulations with opposite initial velocities are conducted from random initial conformations within a transition-state region until they reach the basin minima, which are then assembled to give the distribution of transition paths efficiently. The effects of tRPS are demonstrated using a comparison with direct simulations of protein folding and unfolding, where tRPS is shown to give results consistent with direct simulations and increase the efficiency by up to five orders of magnitude. This approach is generally applicable to stochastic processes with microscopic reversibility, regardless of whether the variables are continuous or discrete.

## 1. Introduction

Thermodynamics and kinetics are two fundamental aspects of physical chemistry. In general, thermodynamics and equilibrium statistics are relatively easy to handle compared to kinetics and nonequilibrium statistics. Based on the ubiquitous Boltzmann distribution, various efficient enhanced sampling methods have been developed to greatly accelerate the molecular simulations of equilibrium properties [1], e.g., umbrella sampling [2,3], the histogram method [4,5], temperature–replica exchange [6,7,8], integrated tempering sampling [9], and metadynamics [10,11]. In recent years, machine learning techniques were also combined with conventional enhanced sampling to explore the vast conformational space of molecules [12,13,14,15,16].

In contrast, there are less known profound principles for kinetics and nonequilibrium statistics. The Onsager reciprocal relations keenly caught the time-reversal symmetry in the underlying microscopic dynamics to express the equality of certain ratios between different pairs of forces and flows [17,18,19]. The Jarzynski equality [20], which revealed an unexpected connection between irreversible work and free-energy difference, actually utilized the invariance of equilibrium distributions. Kinetics was widely described by a transition-state theory [21], which is based only on the information of potential energy surfaces and thus cannot provide an accurate transition rate. For molecular simulations, a direct simulation of transition processes to determine the transition rate is usually inefficient since the simulation trajectory spends most of the time wobbling and swaying in the vicinity of the reactant free-energy minimum, and the transition events to the product basin are extremely rare [22]. A major category of accelerating kinetic simulations was based on a description of the path ensemble where the transition paths can be sampled purposefully [23]. Many simulation methods have been developed accordingly, e.g., transition path sampling (TPS) [24,25,26], transition interface sampling (TIS) [27,28], forward flux sampling (FFS) [29,30], and a Bayesian relation method [31,32,33]. The distribution of the path ensemble is inherently related to the maximum entropy principle [34], and thus, machine learning has potential applications in path statistics and kinetics computation. Other approaches, such as hyperdynamics (using a bias potential to upshift the free-energy basins) [35,36,37], transition path theory [38], reactive flux (Bennet–Chandler estimation of the transmission coefficient to correct the transition-state theory approximation) [39,40], and the aimless shooting algorithm [41], were also explored. Overall, accelerating kinetic simulations often rely on more complex assumptions than enhanced samplings of thermodynamics and are usually less effective.

In this paper, we present a related approach to accelerate kinetic simulation for systems with a free-energy barrier. The approach is established based on the time-reversal symmetry of the microscopic dynamics and the existence of equilibrium distribution and is thus expected to be generally applicable. The obtained formula to calculate the transition rate is bias-free and easy to implement.

## 2. Theoretical Results

**Figure 1:**
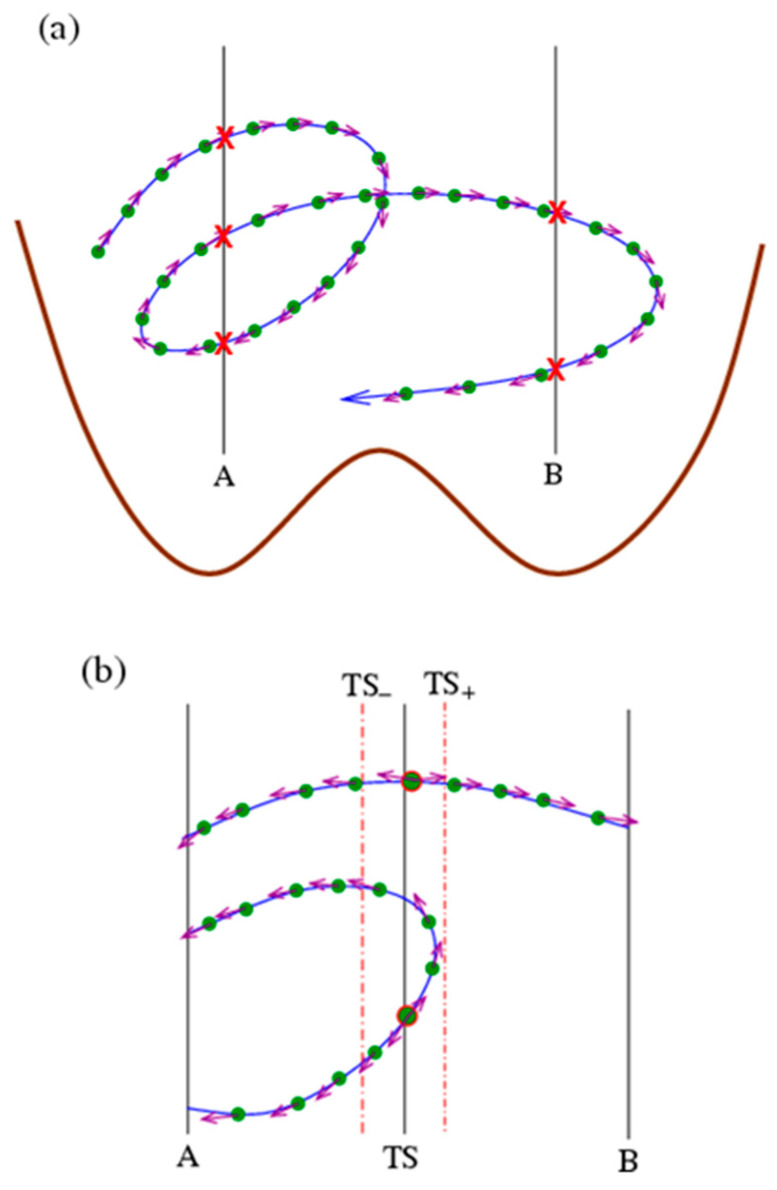
Schematics on accelerating kinetics with time reversibility. (**a**) The construction of the path ensemble. A very long trajectory (blue line) is cut into some short paths (segments) using two cutting planes A and B located at the basin minima of the free-energy profile (brown line). The cutting points are marked with red crossings. Filled green circles represent the conformations sampled on the trajectory with a fixed time step (dt) and violet arrows indicate their velocities. (**b**) A collection of paths crossing the transition state (TS). Starting from an initial conformation (bigger circles with red edge) (obeying equilibrium distribution) within the TS range $\left[TS_{-},TS_{+}\right]$ (indicated by dashed–dotted lines), forward and backward simulations with opposite initial velocities are conducted until they reach any cutting planes (A or B), and, with the time-reversal symmetry, they can be assembled to give a path passing the TS range.

The microscopic state of a system is described by a point in the phase space. Imagine that a phase space has many states (similar to the ensemble picture) that evolve and bifurcate (due to stochastics) over time, forming some infinitely long trajectories. To make practical statistics on trajectories, one should use some methods to cut infinitely long trajectories into paths (segments of trajectories) with a length (duration) that is finite. In the literature, there are two main schemes for cutting trajectories. One is to cut trajectories at a fixed time so that the resulting paths have the same duration. This scheme was adopted by TPS [24,25,26] and S-shooting [42]. Another is to cut trajectories with some fixed planes in the phase space with specified conformational features. For example, for a system with a double-well free-energy profile, the basin minima can be chosen as the cutting point, and the trajectory is cut whenever it crosses the cutting points (Figure 1a). This scheme was adopted in methods such as FFS [29,30], TIS [27,28], and the Bayesian relation method [31,32,33] and was also adopted in this study.

The thermodynamic properties are determined by the state statistics, while the kinetic properties are embedded in the path statistics, i.e., the distribution of paths. To make the analysis more clear, the trajectories and paths are assumed to be composed of a sequence of conformations (plus velocities if necessary) sampled with a very small time step dt (see Figure 1a). Then, a path-i (${𝒫}_{i}$) is described in

(1) $${𝒫}_{i}=\left\{C_{i,j}\right\},\;\;j=1,\;\ldots ,\;L_{i},$$

where $C_{i,j}$ is the j-th conformation of the path ${𝒫}_{i}$, and $L_{i}$ denotes the duration (in a unit of dt) of ${𝒫}_{i}$. The distribution of paths contains the information for state statistics. According to the basic postulates of statistical thermodynamics, the ensemble average (distribution) of a thermodynamic variable is equal to its time average. For a certain property Q (which may present any conformation property, e.g., the fractional number of native contacts for protein folding considered below or the indicator to indicate whether the system falls in a certain free-energy basin A or B), its equilibrium distribution is given by

(2) $$P\left(Q\right)\propto \sum_{i,j}H_{Q}(C_{i,j}),$$

where the characteristic function $H_{Q}\left({\mathrm{Conf}}_{i,j}\right)=1$ or 0, depending on whether $C_{i,j}$ possesses a property Q. In other words, the number of conformations in basin A in the ensemble (at any specified time) is equal to that of the conformations in A for all paths (at various times). When focusing on kinetics, one needs to calculate how many molecules transit from basin A to basin B in a certain time (Figure 1). We use basin minima A and B to cut the trajectory (Figure 1a), and thus, the paths can be classified into four types, depending on whether the beginning and end points are cut by A or B: A–A, A–B, B–A, and B–B. A–B and B–A are transition paths. Notably, each A–B path will contribute a transition event from A to B within dt because each conformation (filled circles in Figure 1) on a trajectory/path will run ahead (evolve) to occupy the position of the preceding one, regardless of the path duration and speeds, which is convenient for calculation. For the transition/reaction

(3) $$A\overset{k}{\to }B,$$

the kinetic equation is

(4) $$\frac{dN_{A}}{dt}=-kN_{A},$$

where $N_{A}$ is the number of molecules in basin A. Therefore, the transition rate coefficient k is calculated as

(5) $$k=-\frac{dN_{A}}{N_{A}dt}=\frac{1}{dt}\frac{\sum_{i}H_{A-B}({𝒫}_{i})}{\sum_{i,j}H_{A}(C_{i,j})},$$

where the characteristic function $H_{A-B}\left({𝒫}_{i}\right)$ indicates whether the path is classified into the type A–B, and $H_{A}\left(C_{i,j}\right)$ indicates whether the conformation falls in the basin A. The denominator in the above equation includes the contributions of paths A–A, A–B, and B–A, but generally, A–A is dominant, and the others can be ignored (unless in the conditions of high temperatures or the shallow wells, where direct simulations can be easily conducted) to yield

(6) $$k=\frac{1}{dt}\frac{\sum_{i}H_{A-B}({𝒫}_{i})}{\sum_{i}L_{i}H_{A-A}({𝒫}_{i})}.$$

Therefore, the kinetics can be readily obtained from path statistics. Equation (6) is theoretically exact, so it is inherently equivalent to other path-based approaches with fixed-plane cuts [27,28,29,30,31,32,33]. But, it is not very useful on its own since direct sampling is usually inefficient, and thus, further ideation is needed as explained below.

In thermodynamics, sampling can be accelerated by applying bias or other means to enhance the sampling probability at certain regions of phase space. Similarly, methods can be developed to enhance the sampling of certain paths in kinetics simulations. Obviously, a direct simulation to generate trajectories/paths to calculate Equation (6) is very inefficient since most produced paths belong to types A–A and B–B but not transition paths A–B and B–A, and the resulting error in the numerator in Equation (6) is large. Then, comes the main idea of this study (Figure 1b), i.e., to enhance the sampling of A–B and B–A paths using the time-reversal symmetry of the microscopic dynamics. Specifically, any A–B path will pass the transition state (TS) (strictly speaking, here we use it not for the genuine transition state but just to refer to a high-free-energy range separating two basins or through which all transition paths have to pass) and will be divided into an A–TS half path and a TS–B half path. The distribution of TS–B half paths can easily be obtained by a shooting simulation from initial states at TS (with an equilibrium distribution that can be obtained by conventional enhanced sampling for equilibrium conformation statistics) and terminated at A or B (to give TS–A and TS–B half paths). Although the A–TS half paths are difficult to obtain in direct simulations from initial states at A, their population is exactly equal to that of the reverse TS–A ones due to the time-reversal symmetry and can be obtained from the shooting simulations from TS. (Strictly speaking, the trajectories generated by MD simulations are stochastic and are thus not necessarily time reversible. The time reversibility of trajectories here should be considered in a more general sense, i.e., that the TS–A paths provide statistically representative examples of the oppositely directed A–TS ones.) Therefore, the difficult uphill path sampling is converted into an easy downhill problem.

In actual simulations, the initial conformations are randomly chosen from an equilibrium distribution within a TS region $\left[TS_{-},TS_{+}\right]$, which are determined via umbrella sampling in the examples below, and forward and backward shooting simulations with opposite initial velocities are conducted until they reach basin minima A or B. Then, the backward half path is reversed and assembled with the forward one to give a path passing the TS range, which may be A–A (i.e., A–TS–A), A–B, B–A, and B–B (i.e., B–TS–B) types. It is noted that such a path may contain more than one conformation in the TS region $\left[TS_{-},TS_{+}\right]$ (the number of which is denoted as $n_{TS}$), which have the same probability to be chosen as the initial state to generate the path. To avoid any double counting of the same path, the sampled paths using shooting simulations should be corrected by a weight of $1/n_{TS}$ to be consistent with those in Equation (6). In addition, it is noted that the denominator of Equations (5) and (6) is actually the equilibrium conformation number in basin A. Taking all these together, it yields

(7) $$k=\frac{1}{dt}\frac{N_{\mathrm{TS}}}{N_{A}}\frac{\sum_{i}H_{A-B}({𝒫}_{i})}{N_{\mathrm{TS}}}=\frac{1}{dt}\frac{N_{\mathrm{TS}}}{N_{A}}\frac{\sum_{i\in S}\frac{1}{n_{\mathrm{TS}}}H_{A-B}({𝒫}_{i})}{\sum_{i\in S}1}=\frac{N_{\mathrm{TS}}}{N_{A}}\frac{\sum_{i\in S}\frac{1}{t_{\mathrm{TS}}}H_{A-B}({𝒫}_{i})}{\sum_{i\in S}1},$$

where $\sum_{i\in S}$ indicates a summation over paths assembled from shooting simulations, and $t_{\mathrm{TS}}=n_{\mathrm{TS}}dt$ is the duration of a path spent within the TS region. $N_{\mathrm{TS}}/N_{A}$ is the population ratio of equilibrium conformations in the TS region and basin A. Equation (7) is the central result of this study, which indicates that the transition rate of kinetics can be obtained from an equilibrium result $N_{\mathrm{TS}}/N_{A}$ and a shooting simulation, both of which are easy to implement. It is applicable to both continuous and discrete variables. We term the method time-reversal path sampling (tRPS).

It is noted that the forward/backward shooting moves with time reversibility were widely employed in previous path samplings [24,25,26,27,28,31,32,33,42], e.g., TPS [24,25,26], TIS [27,28], and the old-fashioned Bennett–Chandler approach [39]. In most cases, they were used as a means to perturb the old paths in order to provide trial paths for Monte-Carlo-like algorithm in constructing the path ensemble slice by slice, and the purpose is to calculate the correlation function or the conditional probability between adjacent slices used in rate formula. In comparison, here we utilized the time reversibility to directly convert the difficult-to-calculate quantity (A–B paths) into an easy-to-calculate quantity (TS–A and TS–B half paths). We did not cast any sampled paths as was carried out in the rejection/acceptance step in MC. We do not need to consider any other intermediate slices except the TS one. Visually speaking, in order to determine the height of the top of the steps, TIS and many closely related methods jump upstairs one by one, while tRPS directly jumps down from the top of the steps to the floor. See Supporting Information for more details.

## 3. Numerical Results

We test the method on the protein-folding problem [43]. Although protein modeling has advanced rapidly over the past 50 years, a direct approach to simulate protein-folding kinetics is still challenging [44]. We consider a well-known model protein, chymotrypsin inhibitor 2 (CI2) with 64 residues (PDB ID: 2CI2), which folds and unfolds in a simple two-state manner [45]. A coarse-grained Gō-like model was adopted to describe the conformational energetics of protein in the folding and unfolding processes [46,47], where the protein conformation is represented by the Cα coordinates of the amino acid residues (see Supporting Information). Molecular dynamic (MD) simulations were conducted to obtain the equilibrium conformation distribution and folding/unfolding rates.

**Figure 2:**
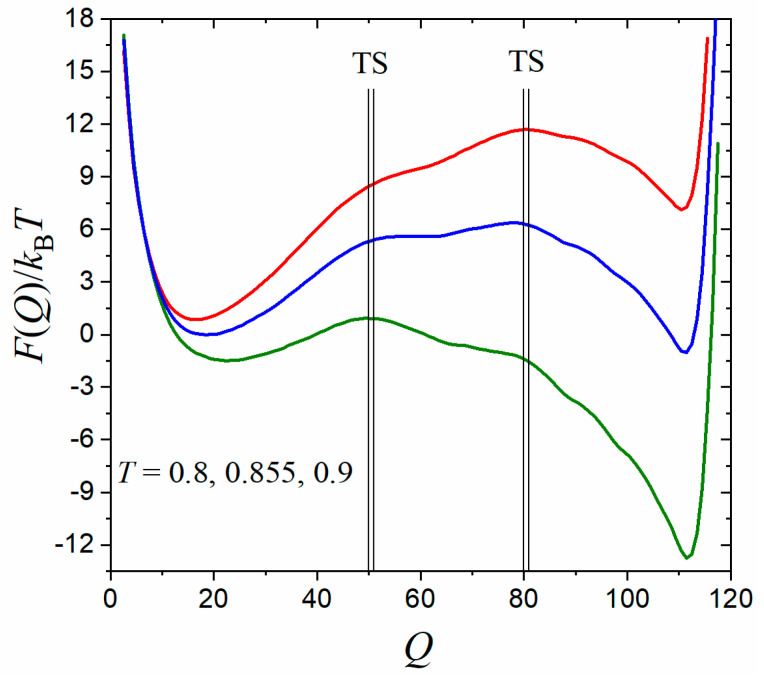
The free-energy profiles of the protein CI2 as a function of the number of native contacts (Q) at different reduced temperatures (from top to bottom): $T=0.9,\;0.855,\;0.8\;\varepsilon /k_{B}$ (where ε is the native contact energy strength). Two choices of transition-state (TS) regions at around Q = 50 and Q=80 are indicated by thin black lines.

The obtained equilibrium free-energy profiles of CI2 using the umbrella sampling technique are plotted in Figure 2 as a function of the number of native contacts (Q). The free-energy profiles exhibit a typical double-well form and are quite smooth, with one basin minimum at Q≈20 for unfolded states and another at Q≈110 for folded states. The folded states become more stable with decreasing the temperature. The midpoint temperature at which folded and unfolded states exhibit equal stability was determined to be T=0.855, where the free-energy barrier is about $6.4\;k_{B}T$. We chose two schemes of TS regions in separating folded/unfolded basins, i.e., $Q_{\mathrm{TS}}\in \left[50,\;51\right]$ and $Q_{\mathrm{TS}}\in \left[80,\;81\right]$ (thin black lines in Figure 2), to calculate the equilibrium population ratio of $N_{\mathrm{TS}}/N_{A}$ (here A can represent unfolded or folded states) as well as preparing initial conformations within the TS region for shooting simulations as required by tRPS in Equation (7).

**Figure 3:**
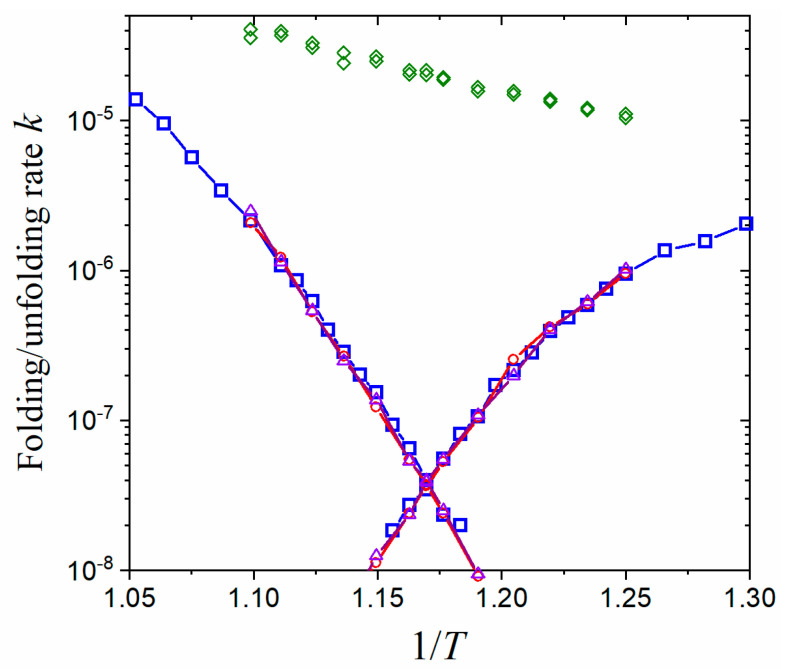
Accelerating folding/unfolding kinetics of CI2 with the tRPS method. Folding (right branches) and unfolding (left branches) rates were measured in a unit of ${\left(\Delta t\right)}^{-1}$, where Δt is the MD time step. The temperature T was measured in a unit of $\varepsilon /k_{B}$. The folding/unfolding rates of direct simulations were plotted in blue squares, while those obtained using tRPS (Equation (7)) were shown in red circles (with $Q_{\mathrm{TS}}\in \left[80,\;81\right]$) and violet triangles (with $Q_{\mathrm{TS}}\in \left[50,\;51\right]$). The sampling rates of transition paths were plotted in scattered green diamonds to demonstrate the acceleration effect. Each datapoint of direct simulations was averaged from about 400 folding/unfolding runs, while those of tRPS were each averaged from about 4000 paths.

The accelerating effect on the kinetics calculation using the tRPS method is demonstrated in Figure 3 with a comparison to direct simulations. The logarithmic folding/unfolding rates form a typical V shape (chevron plot in protein folding). The rates obtained from tRPS agree excellently with the direct simulations in a wide range over two orders of magnitude. It is noted that the choice of TS region, whether $Q_{\mathrm{TS}}\in \left[50,\;51\right]$ or $Q_{\mathrm{TS}}\in \left[80,\;81\right]$, does not affect the agreement since the only requirement for the TS region is that it separates the folded and unfolded basins. It is not required to be the true transition state. As proof of the acceleration, the simulation time consumed in tRPS, e.g., the average time in obtaining a transition path in shooting simulation starting from the TS region (green diamonds in Figure 3), is much shorter than that in direct simulations (blue squares). Although the equilibrium property $N_{\mathrm{TS}}/N_{A}$ is also required by Equation (7), various efficient enhanced sampling methods have been developed previously in obtaining the equilibrium properties (among which we adopted the umbrella sampling here). In addition, the temperature dependence of free-energy difference [$-k_{B}T\ln\left(N_{\mathrm{TS}}/N_{A}\right)$] is approximately linear (Figure S1) and is thus relatively simple to determine.

Usual models of kinetics suggest that the system has to oscillate around the bottom of a basin many times before it finally crosses the barrier to successfully transit into another basin. This is in line with the protein-folding example here: the average duration of A–A and B–B paths remains roughly unchanged with increasing temperature (Figure S2), similar to the characteristics of a simple pendulum. To have one successful A–B or B–A transition, it has to oscillate $10^{3}\sim 10^{6}$ times. The acceleration of tRPS originates from the fact that it does not need to spend a lot of time on the massive oscillations but can directly sample the transition paths.

**Figure 4:**
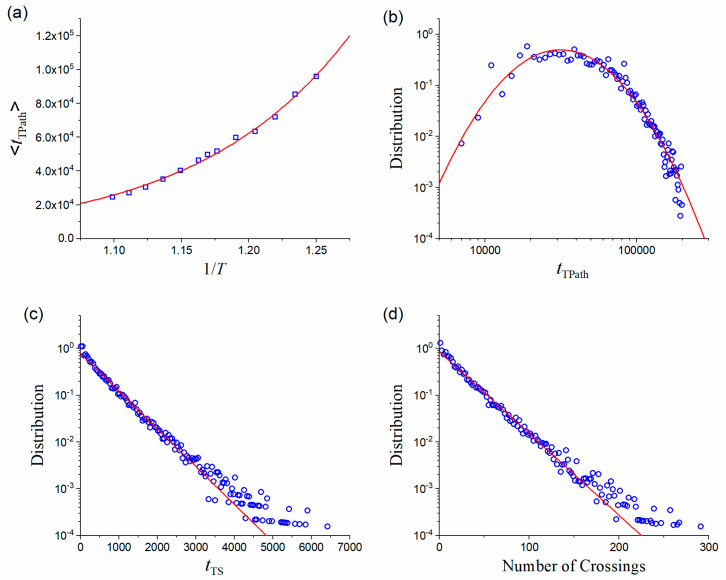
Properties of transition paths. (**a**) The average duration of transition paths ($t_{\mathrm{TPath}}$) as a function of 1/T, which obeys an exponential law (solid line). (**b**–**d**) The distributions of $t_{\mathrm{TPath}}$ (**b**), the duration of a path spent within the TS region ($t_{\mathrm{TS}}$) (**c**) and the number of times a path crosses the TS region (**d**) at the midpoint temperature T=0.855. Solid lines are a quadratic fit in (**b**) and linear fit in (**c**,**d**).

With tRPS, transition paths can readily be obtained for analyses (Figure 4). The average duration of transition paths $\langle t_{\mathrm{TPath}}\rangle$ is in an order of magnitude of $10^{4}$ steps and increases exponentially with decreasing temperature (Figure 4a), much larger than that for A–A and B–B oscillation paths (in the order of magnitude of $10^{2}$ steps, see Figure S2). The logarithmic $t_{\mathrm{TPath}}$ roughly obeys a Gaussian distribution (Figure 4b), similar to previous studies [22,33]. Remarkably, a transition path usually has multiple opportunities to cross the TS region, the number of which well obeys an exponential distribution, i.e., a memoryless distribution (Figure 4d). Consequently, the duration of a path spent within the TS region, $t_{\mathrm{TS}}$, also obeys the exponential distribution (Figure 4c). At the midpoint temperature T=0.855, the average number of crossing times is about 25, and there is an average $t_{\mathrm{TS}}$ of about 540 steps. The large number of crossing times indicates that the conventional transition-state theory would inevitably overestimate the transition rates since it assumes that a transition path crosses the TS region only once. Another discrepancy with the transition-state theory is that the activation enthalpy of folding/unfolding (the minus slope of the unfolding curve in Figure 3 is about 62ε at midpoint temperature) is not equal to the enthalpy difference between the TS region and the unfolded/folded state (contributed by the $N_{\mathrm{TS}}/N_{A}$ term in Equation (7), which is about 72ε for unfolding) due to the contribution of the last term in Equation (7).

**Figure 5:**
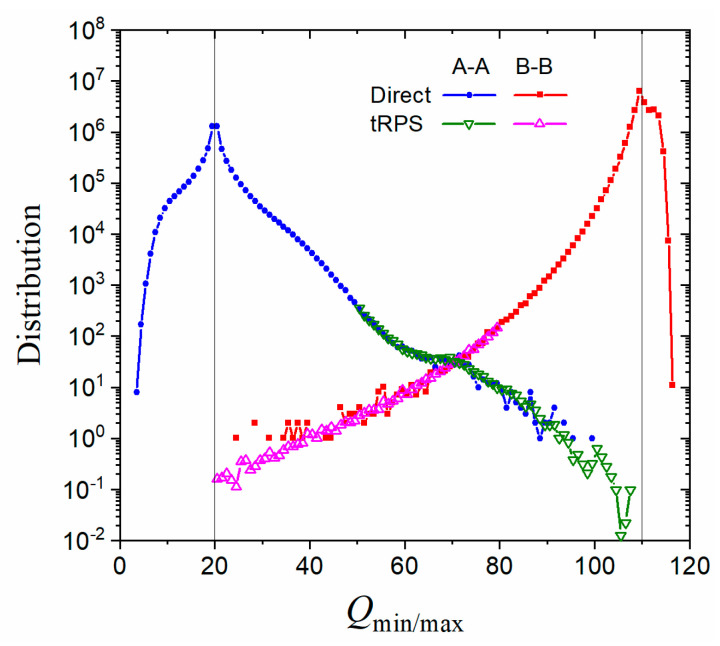
Distribution of minimal/maximal Q for A–A and B–B paths at T=0.855. $Q_{A}=20$ and $Q_{B}=110$ were used in cutting paths. Filled symbols represent datapoints from direct simulations, while open symbols for those from tRPS with $Q_{\mathrm{TS}}\in \left[50,\;51\right]$ (down triangles) and $Q_{\mathrm{TS}}\in \left[80,\;81\right]$ (up triangles).

The tRPS method can be combined with direct simulations to provide much more comprehensive results. For example, the minimal/maximal Q value of a path can be adopted to measure how far it can go, and the resulting path population decreases exponentially with the distance between $Q_{\min/\max}$ and the cutting point (Figure 5). This makes the paths with distant $Q_{\min/\max}$ hard to sample in direct simulations. The tRPS method, on the other hand, samples only the paths that cross the TS region and thus possesses the capability to probe distant $Q_{\min/\max}$. The patches they provided can be combined to give a smooth and complete distribution (Figure 5).

**Figure 6:**
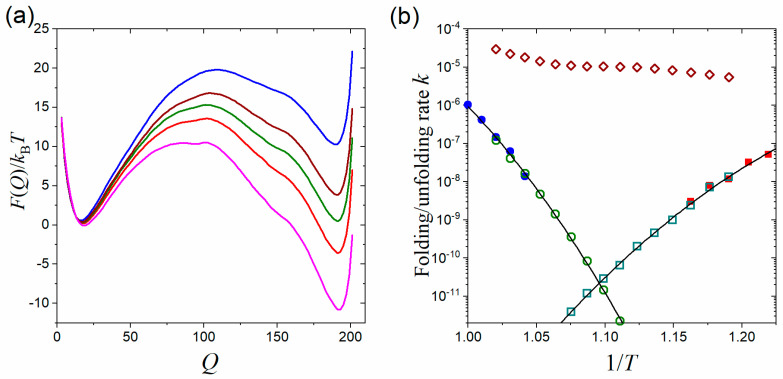
Equilibrium thermodynamics and kinetics of protein folding/unfolding for acylphosphatase. (**a**) The free-energy profiles at different reduced temperatures (from top to bottom): $T=0.95,\;0.925,\;0.913,\;0.9,\;0.875\;\varepsilon /k_{B}$. (**b**) Accelerating kinetics with tRPS. The temperature T was measured in a unit of $\varepsilon /k_{B}$. The folding/unfolding rates of direct simulations were plotted in filled squares/circles, while those obtained using tRPS were shown in open squares/circles (with $Q_{\mathrm{TS}}\in \left[100,\;102\right]$). The sampling rates of transition paths were plotted in scattered diamonds to demonstrate the acceleration effect.

Transition rates decay exponentially with the barrier height, but the duration of transition paths usually depends on the barrier in a much weaker logarithmic law [22,48]. This makes tRPS even more powerful when the barrier is high. As proof, we apply tRPS on another protein, acylphosphatase (PDB ID: 1APS) with 98 residues, which was listed as a slow-folding protein in a previous study [49]. The obtained free-energy profile is smooth, possessing a high barrier of about $15\;k_{B}T$ at a midpoint temperature of T=0.913 (Figure 6a). The folding/unfolding is slow and extremely difficult to determine with direct simulations. Therefore, we conducted direct simulations only in some feasible temperature ranges (filled circles/squares in Figure 6b) and applied tRPS to complete the gaps (open circles/squares). The results of direct simulation and tRPS in the overlapping area are well consistent with each other. The data combine to give a nice chevron plot. As a main expense of tRPS, the average duration (open diamonds in Figure 6b) of transition paths for acylphosphatase is similar in order of magnitude to that for CI2. The increase in the efficiency of kinetic calculation using tRPS is up to five orders of magnitude around the midpoint temperature if not taking into account the expense of equilibrium calculations (for $N_{\mathrm{TS}}/N_{A}$ in Equation (7)).

## 4. Discussion

The validity of tRPS relies on the time reversibility. Although it cannot be applied to irreversible systems as methods like FFS [29], it possesses the benefits of “shooting from the top” [33] to avoid the possible inadequate choice of initial states in the basin that are capable/incapable of crossing the barrier. Although the TS region was not particularly optimized in our examples (Figure S3), the resulting rates from tRPS are satisfactory (Figure 3 and Figure 6). Another underlying assumption of tRPS is the pre-equilibrium after a transition path; i.e., after crossing the cutting planes at the basin bottom, the system will pre-equilibrate within the basin but not cross the barrier back to the reactant basin soon. This can be tested by extending the shooting simulation after the path hits the cutting planes. Analyses of the examples of CI2 and acylphosphatase show that the error caused by the preequilibrium assumption is negligible. In addition, Equation (7) is beneficial in terms that it is less affected by possible complicated energy landscapes around the bottom of basins. For the case of acylphosphatase, some abnormal high durations of transition paths were observed in shooting simulations at low temperatures (Figure S4), likely caused by hidden traps within basins, but the resulting kinetic rates seem unaffected (Figure 6).

Under harsh conditions such as hysteresis, the efficiency of tRPS may drop dramatically. This is a challenge that most path-based methods encounter. The procedure of tRPS contains two parts: equilibrium sampling of the TS region and dynamic shooting simulations from the initial conformations obtained in the TS region. In this study, the TS region was preassigned manually. In principle, a self-adaptive TS region for the purpose of efficiency optimization can be designed by allowing mutual interplays between two parts; i.e., let the shooting results also conversely affect the choice of TS region. Enhanced sampling methods and machine learning may play a role in it.

## 5. Conclusions

In this paper, we have proposed a method to accelerate the simulations for determining the kinetics of systems. The approach was constructed based on the time reversibility of microscopic dynamics and is thus generally applicable. It is easy to implement and can operate on both continuous and discrete variables. The method was tested on the folding/unfolding of two proteins with fast and slow kinetics. In areas where direct kinetic simulations can be readily performed, the accelerating method produced results fully consistent with direct simulations. In areas where direct simulations are inaccessible, the accelerating method provided reasonable results at little cost, with an increase in efficiency up to five orders of magnitude. The technique is easily applied to other kinds of calculations, such as quantum dynamics and chemical reactions.

## Data Availability

Data are contained within the article and supplementary materials.

## Supplementary Materials

The following supporting information can be downloaded at https://www.mdpi.com/article/10.3390/molecules28248147/s1: the native-centric Gō-like model; molecular dynamics simulations; a theoretical comparison with TIS; Figure S1: the temperature dependence of free-energy difference; Figure S2: some path properties determined from direct simulations; Figure S3: the path number sampled in tRPS; and Figure S4: the duration of shooting paths from the TS region for acylphosphatase. References [50,51,52] are cited in Supplementary Materials.
