# Supplementary material for: Accelerating Kinetics with Time-Reversal Path Sampling
Source: Molecules. 2023 Dec 18;28(24):8147. doi: 10.3390/molecules28248147 (PMC10745403; doi:10.3390/molecules28248147)
Supplement: Supplementary file 1 [file molecules-28-08147-s001.zip › molecules-2753116-supplementary.pdf]

SUPPORTING INFORMATION

Accelerating Kinetics with Time-Reversal Path Sampling

Zhirong Liu*

College of Chemistry and Molecular Engineering, and Beijing National Laboratory for Molecular Sciences (BNLMS), Peking University, Beijing 100871, China; liuzhirong@pku.edu.cn

MATERIALS AND METHODS

Native-centric Gō-like model

We adopted a coarse-grained Gō-like model to describe the energetics of proteins in the folding and unfolding processes.[46, 47, 50] The protein conformation is represented by the Cα coordinates of the amino acid residues. Only native interactions are favorable in the model. The total potential energy of the model was given as

$$\begin{aligned} V_{\text{total}} &= V_{\text{stretching}} + V_{\text{bending}} + V_{\text{torsion}} + V_{\text{non-bonded}} \\ &= \sum_{\text{bonds } i} K_{\text{r}} \left(r_i - r_i^{(0)} \right)^2 + \sum_{\text{angles } i} K_\theta \left(\theta_i - \theta_i^{(0)} \right)^2 \\ &\quad + \sum_{\text{dihedrals } i} \left\{ K_\phi^1 \left[1 - \cos\left(\phi_i - \phi_i^{(0)} \right) \right] + K_\phi^3 \left[1 - \cos\left(3\phi_i - 3\phi_i^{(0)} \right) \right] \right\} \\ &\quad + \sum_{i<j-3}^{\text{native}} U\left(r_{ij}; r_{ij}^{(0)}, \varepsilon \right) + \sum_{i<j-3}^{\text{nonnative}} \varepsilon \left(\frac{r_{\text{rep}}}{r_{ij}} \right)^{12}, \end{aligned} \tag{S1}$$

where r_i, θ_i, ϕ_i and r_{ij} are the virtual bond length, bond angle, torsion angle and nonbonded spatial distance defined by Cα atom positions, respectively. $r_i^{(0)}$, $\theta_i^{(0)}$, $\phi_i^{(0)}$ and $r_{ij}^{(0)}$ are the corresponding native values available from the PDB structure. $U\left(r_{ij}; r_{ij}^{(0)}, \varepsilon\right)$ is a Lennard-Jones-like attraction potential with an extra solvation/desolvation barrier,[46, 50] which applies only for residue pairs in the native contact set. $U\left(r_{ij}; r_{ij}^{(0)}, \varepsilon\right)$ has a potential minimal $U = -\varepsilon$ at $r_{ij} = r_{ij}^{(0)}$. A pair of residues is defined to be in the native contact set if they are separated by at least three residues and a pair of their non-hydrogen atoms are less than 4.5 Å apart in the native PDB structure. $\varepsilon\left(\frac{r_{\text{rep}}}{r_{ij}}\right)^{12}$ is a nonnative repulsive term for any residue pairs being not in the native contact set. For CI2, the truncated form is composed of 64 residues,[45] and the PDB ID of its solved native structure is 2CI2. The resulting number of contacts in the native structure is $Q^{(\text{N})} = 131$. A second protein considered in simulations, acylphosphatase (PDB ID: 1APS), contains 98 residues and $Q^{(\text{N})} = 229$. Parameters of the model were set $r_{\text{rep}} = 4.0$ Å, $K_{\text{r}} = 100\varepsilon$, $K_\theta = 20\varepsilon$, $K_\phi^1 = \varepsilon$, $K_\phi^3 = 0.5\varepsilon$ with interaction strength $\varepsilon = 1.0\varepsilon_0$ (where ε_0 is a reference energy scale) as have been used previously.[47, 50]

Molecular dynamics simulations

Simulations were performed in the form of Langevin dynamics:[47, 51]

$$m\frac{d}{dt}\mathbf{v}(t) = \mathbf{F}_{\text{conf}}(t) - m\gamma\mathbf{v}(t) + \boldsymbol{\eta}(t), \tag{S2}$$

where m, $\mathbf{v}$, $\mathbf{F}_{\text{conf}}$, γ and $\boldsymbol{\eta}$ are mass, velocity, conformational force, friction (viscosity) constant and random force, respectively. The conformational force is equal to the negative gradient of the total potential energy V_{total} in Eq. (S1).

The time scale of simulations is controlled by the quantity $\tau = \sqrt{ma^2/\varepsilon_0}$, with the length scale $a = 4$ Å and a reference energy scale $\varepsilon_0 = 1$. The friction constant lies in the overdamped region with $\gamma = 1.0\tau^{-1}$. The molecular dynamics time step is set to be $\Delta t = 0.005\tau$. Simulation times in this study are presented in units of Δt. The temperature T is given in a reduced form with a unit of ε/k_B. The coordinates are measured in units of Å.

The free-energy profiles as functions of number of native contact (Q) were obtained using the umbrella sampling method, and a smoothed version of Q was adopted to facilitate the calculation of forces for umbrella bias potential.[37] The force constant of bias potential is $0.015\varepsilon_0$ for CI2 and $0.01\varepsilon_0$ for acylphosphatase. Typically, 8×10^7 time steps were used in simulations to obtained equilibrium conformation statistics for each bias potential.

Umbrella sampling was also used in preparing initial conformations within specified range of Q for direct folding/unfolding simulations and the accelerating method of time-reversal path sampling (tRPS).

At each temperature, the folding/unfolding rate obtained by direct simulations was averaged from about 400 folding/unfolding runs, i.e., from 400 random initial conformations within the unfolded/folded basin. The rate obtained by tRPS was each averaged from about 4000 paths, i.e., a forward and a backward shooting simulations with opposite initial velocities were conducted from each of 4000 random initial conformations within a transition-state region.

Theoretical comparison with TIS

We first make a brief introduction on TIS and one of its variants, the replica exchange TIS (RETIS), based on the references[27, 28, 52]. The reaction rate was written as[27, 28, 52]

$$k_{AB} = f_A P(\lambda_B|\lambda_A) = f_A \prod_{i=0}^{n-1} P_A(\lambda_{i+1}|\lambda_i), \tag{S3}$$

where the phase space is divided by a set of n interfaces (slices) $\{\lambda_A = \lambda_0, \ldots, \lambda_i, \ldots, \lambda_n = \lambda_B\}$. f_A is the flux of paths through the initial interface $\lambda_A = \lambda_0$ per unit time. $P(\lambda_B|\lambda_A)$ is a conditional probability that a path starting from A, after having crossed λ_A, will cross the interface λ_B before returning to λ_A. It can be conveniently factorized into the probabilities $P_A(\lambda_{i+1}|\lambda_i)$ that a path starting from A crosses the interface λ_{i+1} after having crossed the interface λ_i without returning to A first. Essential to construct the path ensembles (that crossed each interface λ_i) is to utilize MC-like moves. The most widely adopted MC move is the shooting move. In this move, a time slice of the last accepted path $\mathbf{x}^{(\mathrm{o})}$ is taken at random. Then, this point is modified, for instance, by changing the velocities of this point. Finally, this new point is used to shoot forward and backward in time using MD simulations in order to create a new (trial) path $\mathbf{x}^{(\mathrm{n})}$. The acceptance rule of the move for MC algorithm can be written as[28]

$$P_{\mathrm{acc}}\left[\mathbf{x}^{(\mathrm{o})} \to \mathbf{x}^{(\mathrm{n})}\right] = \hat{h}(\mathbf{x}^{(\mathrm{n})})\min\left[1, \frac{P[\mathbf{x}^{(\mathrm{n})}]}{P[\mathbf{x}^{(\mathrm{o})}]} \frac{P_{\mathrm{gen}}[\mathbf{x}^{(\mathrm{n})} \to \mathbf{x}^{(\mathrm{o})}]}{P_{\mathrm{gen}}[\mathbf{x}^{(\mathrm{o})} \to \mathbf{x}^{(\mathrm{n})}]}\right], \tag{S4}$$

where $\hat{h}(\mathbf{x}^{(\mathrm{n})})$ is 1 if the new path fulfills the path ensemble's condition (e.g., crossing λ_i), otherwise it is 0. P_{gen} is the generation probability. If accepted, replace the old path with the new (trial) one; otherwise, cast the new (trial) path and keep the old one. Through this method, path ensembles can be constructed slice-by-slice, i.e., utilized the ensemble with paths crossing λ_i to construct another ensemble with paths crossing λ_{i+1}, and calculate $P_A(\lambda_{i+1}|\lambda_i)$ in Eq. (S3).

From the above description, it can be known that the forward/backward shooting moves with time-reversibility were used in TIS/RETIS to perturb the old path into new (trial) path which was further fed into a Monte-Carlo-like algorithm of Eq. (S4). The new path inevitably shares some similarity with the old one. In other words, they are close in the path space. In some cases, the new (trial) path will be cast according to Eq. (S4).

In contrast, in our tRPS method, the time-reversibility was utilized to directly convert the difficult-to-calculate quantity (A-B paths, which contain A-TS or B-TS half-paths) into easy-to-calculate quantity (TS-A and TS-B half-paths). It is not necessary to consider a series of interfaces (slices). The forward/backward shooting was started from a conformation randomly chosen from the equilibrium distribution within the TS region $[TS_-, TS_+]$, but not from the previously obtained paths as done in TIS and RETIS. This is more efficient since equilibrium distribution is more easy to obtained (where enhanced sampling can be used), and the results paths in tRPS are uncorrelated, i.e., they are not necessarily close in the path space. In addition, no Monte-Carlo-like algorithm and acceptance/rejection step was used. Visually speaking, in

order to determine the height of the top of the steps, TIS and many closely related methods jump up stairs one by one, while tRPS directly jumps down from the top of the steps.

For another approach, S-shooting[42], although a TS region is used without other slices, forward/backward shooting was still started from a conformation randomly chosen from the previously obtained paths as done in TIS and RETIS. The shooting points do not necessarily locate within the TS region. So it is different from tRPS.

SUPPLEMENTARY FIGURES

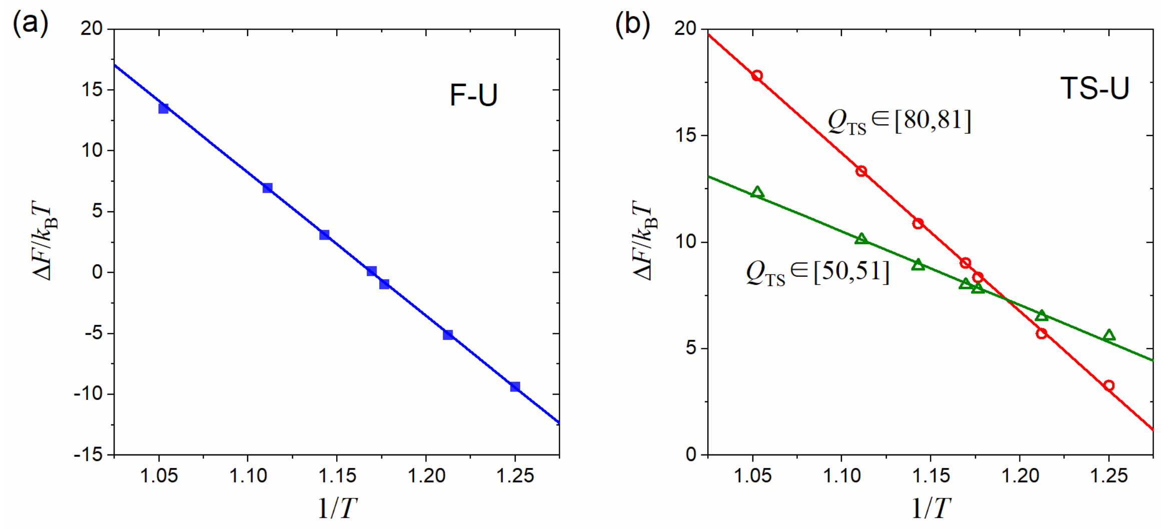

Figure S1. The temperature-dependence of free energy difference between: (a) folded and unfolded states [$\Delta F = -k_BT\ln(\frac{N_F}{N_U})$], (b) transition states and unfolded states [$\Delta F = -k_BT\ln(\frac{N_{TS}}{N_U})$]. The transition state region was defined as $Q_{TS} \in [50,51]$ (green) or $Q_{TS} \in [80,81]$ (red) in panel (b). Solid lines are linear fits to the data points.

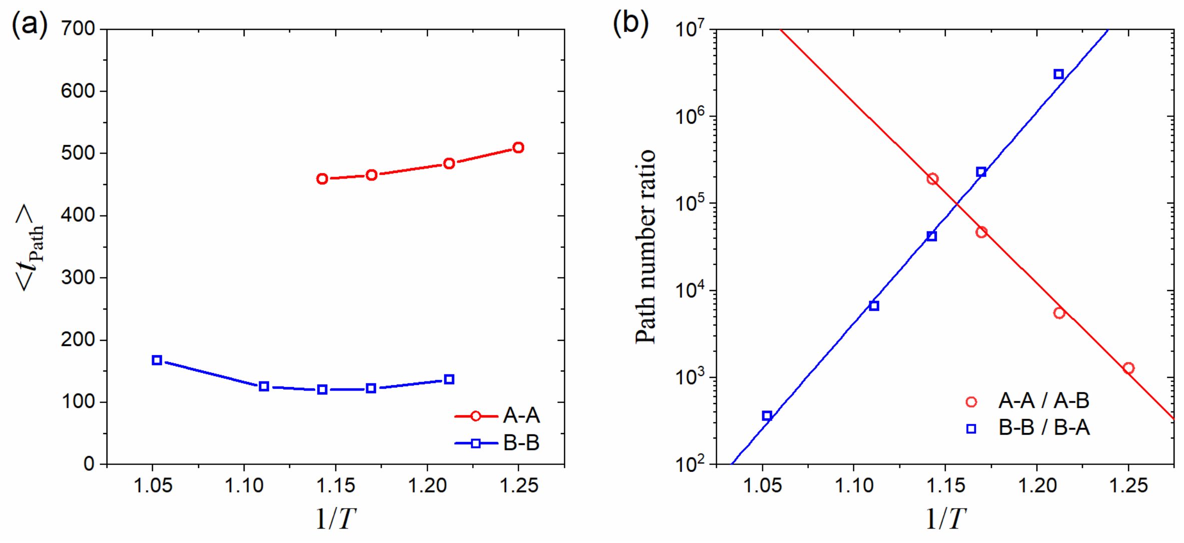

Figure S2. Some path properties determined from direct simulations. (a) The averaged duration of A-A and B-B paths (t_{Path}) as a function of $1/T$. A is unfolded states, being relatively loose; B is folded state, which is relatively compact and possesses a higher vibration frequency. t_{Path} of A-A and B-B is correlated with their vibration period, so t_{Path} of A-A is larger than that of B-B. (b) The path number ratio between A-A and A-B (or B-B and B-A) paths, where solid lines are linear fits. $Q_A = 50$ and $Q_B = 110$ were used in cutting paths.

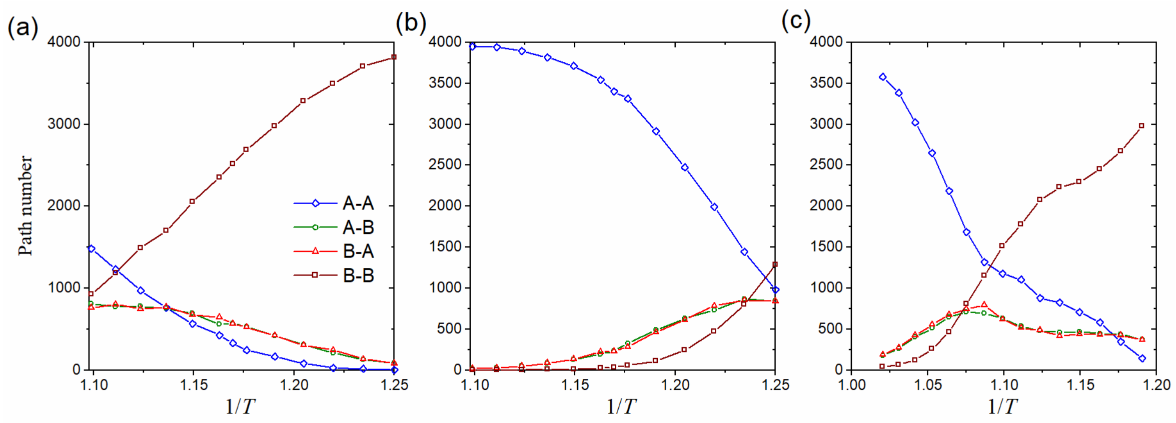

Figure S3. Path number sampled in tRPS for (a) CI2 with $Q_{\mathrm{TS}} \in [80,81]$, (b) CI2 with $Q_{\mathrm{TS}} \in [50,51]$, and (c) acylphosphatase with $Q_{\mathrm{TS}} \in [100,102]$.

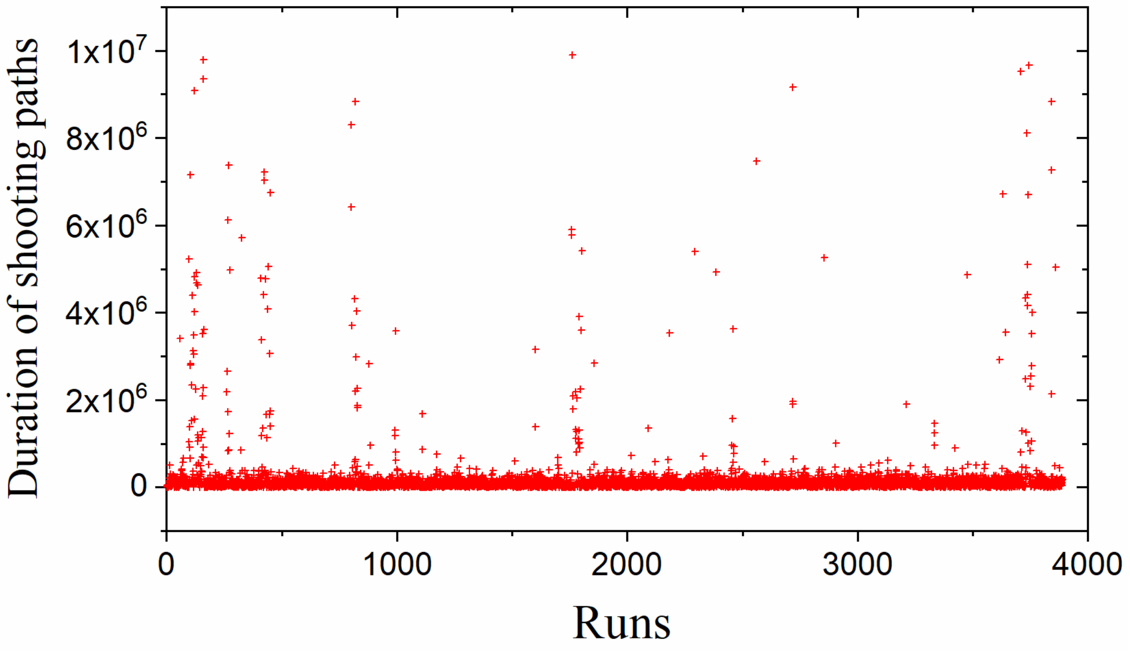

Figure S4. Duration of shooting paths from TS region for acylphosphatase at $T = 0.82$. The abnormal high values of duration are likely caused by traps inside the free-energy basins.
